# Antimicrobial resistance and integrons of commensal *Escherichia coli* strains from healthy humans in China

**DOI:** 10.1179/1973947813Y.0000000113

**Published:** 2014-06

**Authors:** Bin Li, Zhi-chang Zhao, Mei-hua Wang, Xin-hong Huang, Yu-hong Pan, Ying-ping Cao

**Affiliations:** 1 Department of Clinical Laboratory, Fujian Medical University Union Hospital, Fuzhou, Fujian, China; 2 Department of Pharmacy, Fujian Medical University Union Hospital, Fuzhou, Fujian, China

Antibiotic therapy can affect not only the pathogenic bacteria, but also commensal microorganisms in the gut flora of humans, which might serve as a reservoir of antimicrobial resistance genes.^1^ *Escherichia coli* can be found widely in nature, and can also be implicated in human infectious diseases. Therefore, fecal *E. coli* is often considered as a good indicator for selection pressure imposed by antimicrobial use.^2^ Recently, many researchers began showing interest in the surveillance of antimicrobial resistance in normal flora of humans.^3^–^5^ In China, however, very little is known about the prevalence of antibiotic resistant genes in intestinal tracts of healthy people. The aim of our study is to investigate the prevalence of antimicrobial resistance genes and diversity of integrons in fecal *E. coli* strains recovered from healthy persons in China.

In 2009, we performed a research on the fecal *E. coli* strains isolated from asymptomatic healthy unrelated human subjects in Fuzhou, China.^6^ And in this study, 150 fecal *E. coli* strains were randomly selected from those that were collected in 2009. *E. coli* ATCC 25922 was used as a positive control.

Susceptibility to antibiotics was assessed by the disc diffusion method according to the CLSI.^7^ The following 18 antimicrobial agents were tested in this study: ampicillin (AMP, 10 μg), ampicillin plus sulbactam (SAM, 10/10 μg), cefazolin (CZO, 30 μg), cefuroxime (CXM, 30 μg), ceftazidime (CAZ, 30 μg), cefotaxime (CTX, 30 μg), cefepime (FEP, 30 μg), cefoxitin (FOX), aztreonam (ATM, 30 μg), imipenem (IPM, 10 μg), amikacin (AMK, 30 μg), kanamycin (KAN, 30 μg), streptomycin (STR, 10 μg), nalidixic acid (NA), ciprofloxacin (CIP, 5 μg), trimethoprim-sulfamethoxazole (SXT, 1.25/23.75 μg), chloramphenicol (CHL, 30 μg), and tetracycline (TET, 30 μg). The discs were obtained from Oxoid Ltd (Basingstoke, Hampshire, England).

The presence of genes associated with ampicillin (*bla*_TEM_, *bla*_SHV_, *bla*_OXA-1_), tetracycline (*tetA*–*tetE*), streptomycin (*aadA*), sulphonamide (*sul1*, *sul2* and *sul3*), kanamycin (*aphA1* and *aphA2*), chloramphenicol resistance (*cmlA* and *floR*), and plasmid-borne quinolone resistance genes (*qnrA*, *qnrB*, and *qnrS*) was also analyzed by PCR.^8^,^9^ The presence of class 1, 2, and 3 integrons was characterized by PCR in all *E. coli* strains.^8^ The characterization of the variable region of class 1 and 2 integrons was performed by PCR and subsequent DNA sequencing.

As shown in Table 1, the resistances to STR (79.3%), NA (53.3%), TET (50%), AMP (44%), and SXT (32%) were higher than those of the other antimicrobials (0–19.3%). Of these, streptomycin and tetracycline have been widely used as growth promoters or prophylactic agents in animal husbandry.^10^ The high resistance of gut flora may be due to the extensive and long-term use of these antibiotics in humans and in livestock. Our study also found that the resistance rate of human fecal *E. coli* strains to third-generation cephalosporins such as CTX (15.3%) and CAZ (4.0%) was higher than that in other countries.^11^,^12^ In China, beta-lactams, aminoglycosides, and quinolones are often used to cure *Enterobacteriaceae* infections. Therefore, it is not surprising that the high resistance of commensal flora may be due to the extensive use of these antimicrobials.

**Table 1:** Association between antibiotic profile and integrons in 150 fecal *E.coli* strains*^a^*

Antibiotic	Antibiotic susceptibility									
	All isolates (*n* = 150)			Integron-positive isolates (*n* = 40)			Integron-negative isolates (*n* = 110)			*P*-value
	% R	% I	% S	% R	% I	% S	% R	% I	% S	
AMP	44	4	52	65	2.5	32.5	36.4	4.5	59.1	0.006
SAM	7.3	14.7	78	17.5	25	57.5	3.6	10.9	85.5	0.001
CZO	16.7	44.7	38.7	17.5	47.5	35	16.4	43.6	40	0.856
CXM	14	0.7	85.3	15	0	85	13.6	0.9	85.5	0.851
CAZ	4	4	92	2.5	5	92.5	4.5	3.6	91.8	0.884
CTX	15.3	0.7	84	15	0	85	15.5	0.9	83.6	1.000
FEP	3.3	2	94.7	5	2.5	92.5	2.7	1.8	95.5	0.678
FOX	1.3	0.7	98	0	2.5	97.5	1.8	0	98.2	0.319
ATM	4	0.7	95.3	7.5	0	92.5	2.7	0.9	96.4	0.518
IPM	0	0	100	0	0	100	0	0	100	—
AMK	2	10.7	87.3	2.5	15	82.5	1.8	9.1	89.1	0.480
KAN	14.7	43.3	42	30	45	25	9.1	42.7	48.2	0.002
STR	79.3	20	0.7	90	10	0	75.5	23.6	0.9	0.116
NA	53.3	3.3	43.3	70	2.5	27.5	47.3	3.6	49.1	0.041
CIP	19.3	2	78.7	27.5	2.5	70	16.4	1.8	81.8	0.226
SXT	32	2	66	62.5	5	32.5	20.9	0.9	78.2	<0.001
CHL	11.3	0	88.7	32.5	0	67.5	3.6	0	96.4	<0.001
TET	50	1.3	48.7	75	2.5	22.5	40.9	0.9	58.2	<0.001

*^a^* Abbreviations:%R, resistant rate;%I, intermediate rate;%S, susceptible rate; *n*, total number of isolates; AMP, ampicillin; SAM, ampicillin/sulbactam; CZO, Cefazolin; CXM, Cefuroxime; CAZ, ceftazidime; CTX, Cefotaxime; FEP, cefepime; FOX, cefoxitin; ATM, aztreonam; IPM, imipenem; AMK, amikacin; KAN, kanamycin; STR, streptomycin; NA, nalidixic acid; CIP, ciprofloxacin; SXT, trimethoprim-sulfamethoxazole; CHL, chloramphenicol; TET, tetracycline.

Of the 150 *E. coli* strains tested, 40 (26.7%) carried integrons, among which 39 (97.5%) carried *intI1* and only 1 strain carried *intI2*. Class 3 integrons were not detected in the study. The prevalence of the integrase gene (26.7%) in this study was similar to that in Spain (29%),^11^ however, it is lower than that found in clinical *Enterobacteriaceae* isolates in China (59.9%).^13^ All integron-positive strains showed resistance to at least three antimicrobial agents, as found in previous studies.^11^,^13^,^14^

Six different gene cassette arrangements were demonstrated in 24 of the 39 *intI1*-positive isolates and were as follows (number of isolates): *dfrA17*-*aadA5* (8), *aadA1*(6), *aadA2*(4), *aadA5*(2), *dfrA5*(2), and *dfrA7*(2). Only one *E. coli* isolate in this study containing class 2 integrons presented the gene cassette array in its variable region: *dfrA1- sat2- aadA1*, also being frequent in other study.^8^ And the most frequently detected resistance genes (*aadA* family and *dfr* family) in this study were aminoglycoside adenylyltransferase genes and dihydrofolate reductase genes that confer resistance to streptomycin, spectinomycin, and trimethoprim. The high frequencies of *aadA* and *dfr* family detection in integrons are similar to other studies.^11^,^13^,^14^

Integrons were significantly associated with AMP, SAM, SXT, KAN, NA, CHL, and TET (Table 1), and all 40 integron-positive isolates showed resistance to at least three antimicrobial agents. Higher percentages of resistance to some antimicrobial agents (STR, AMP, TET, SXT, CHL, and NA) were observed among integron-positive isolates with respect to integron-negative isolates. Table 2 shows the antimicrobial resistance genes detected in our isolates. The *bla*TEM gene was detected in 27 of the 74 ampicillin-resistant isolates and the *bla*OXA-1 gene in one additional isolate. Regarding tetracycline resistance, the *tetA* gene was the predominant one in those strains, and it has been previously suggested that *tetA* gene and class 1 integrons are often located on the same conjugative plasmid.^15^

**Table 2:** Genes of resistance detected among commensal *E. coli* strains with and without integrons

Antibiotics	Integron-positive strains (*n* = 40)		Integron-negative strains (*n* = 110)	
	number	Genes detected (no. of strains)	number	Genes detected (no. of strains)
ampicillin	27	*bla*TEM (12)	45	*bla*TEM (15)
		*bla*OXA-1 (1)		
tetracycline	31	*tetA* (5)	46	*tetA* (10)
		*tetB* (5)		*tetB* (7)
		*tetA*+*tetB* (1)		
sulponamide	27	*sul-1* (3)	24	*sul-1* (7)
		*sul-2* (1)		*sul-1*+ *sul-2* (5)
		*sul-1*+ *sul-2* (8)		
		*sul-2*+ *sul-3* (1)		
nalidixic acid	29	*qnrS* (1)	55	ND^a^
streptomycin	33	*aadA* (8)	61	ND^a^
kanamycin	19	*aphA1* (7)	31	*aphA1* (1)
chloramphenicol	13	*cmlA1* (6)	4	*cmlA1* (2)
		*floR* (2)		*floR* (1)

^a^ ND, no gene detected.

Higher percentage of resistance to some antimicrobial agents (aminoglycoside, sulphanilamides, quinolones, and beta-lactam agents) were observed among integron-positive strains with respect to integron-negative strains (Table 1). The fact could be explained by the presence of resistance genes in the conserved or variable region of integrons, or by the inclusion of resistance genes in the same mobile elements that carry integrons.^11^

The following genes were identified among 51 SXT-resistant isolates (integron-positive/integron-negative isolates): *sul1* (3/7), *sul2* (1/0), *sul1*+*sul2* (8/0) and *sul2*+*sul3* (1/0). The *aphA1* gene was found in 8 of 50 kanamycin-resistant *E. coli* isolates. Among the 17 chloramphenicol-resistant *E. coli* isolates, the *cmlA* and *floR* genes were found in eight and three isolates, respectively. Among the 29 nalidixie acid-resistant integron-positive isolates, the *qnrS* gene was found in one isolate. And *aadA* gene was detected in 8 of 33 streptomycin resistant integron-positive isolates.

In conclusion, this study demonstrates that multidrug resistance genes and integrons are widespread in fecal *E. coli* strains from healthy people in China. The integrons may contribute to the dissemination of antibiotic resistance in the gut flora. The data of this study provides useful information regarding the dissemination of antibiotic resistance among healthy humans in the community. Continued surveillance of normal flora should be carried out to predicting the antimicrobial resistance trends of clinical *Enterobacteriaceae* isolates in this region.
